# Video-based evaluation of infant crawling toward quantitative assessment of motor development

**DOI:** 10.1038/s41598-020-67855-0

**Published:** 2020-07-09

**Authors:** Katsuaki Kawashima, Yasuko Funabiki, Shino Ogawa, Hideaki Hayashi, Zu Soh, Akira Furui, Ayumi Sato, Taiko Shiwa, Hiroki Mori, Koji Shimatani, Haruta Mogami, Yukuo Konishi, Toshio Tsuji

**Affiliations:** 1 Graduate School of Engineering, Hiroshima University, 1-4-1 Kagamiyama, Higashi-Hiroshima, Hiroshima 739-8527 Japan; 2 Graduate School of Human and Environmental Studies, Kyoto University, Yoshida-Nihonmatsu-Cho, Sakyo-Ku, Kyoto, 606-8507 Japan; 3 Faculty of Information Science and Electrical Engineering, Kyushu University, 744 Motooka, Nishi-Ku, Fukuoka, 819-0395 Japan; 4 Future Robotics Organization, Waseda University, 1-104 Totsukamachi, Shinjuku-Ku, Tokyo, 169-8050 Japan; 5 Department of Physical Therapy, Prefectural University of Hiroshima, 1-1 Gakuen, Mihara, Hiroshima 723-0053 Japan

**Keywords:** Paediatrics, Signs and symptoms

## Abstract

This study proposes a quantitative evaluation support system for infant motor development and uses the system to analyze hands-and-knees creeping and belly crawling. This system measures movements using two video cameras and extracts movement features via background and inter-frame subtractions of original images. Eight evaluation indices for each crawling cycle are calculated, enabling markerless movement analysis of infants. Cross-sectional analysis of 16 10-month-olds confirmed significant differences between hands-and-knees creeping and belly crawling in five of the eight indices, demonstrating the system capability to quantitatively differentiate between creeping and crawling. Longitudinal analysis of one infant (aged 7–10 months) also suggested that the proposed quantitative indices can follow changes in crawling characteristics and evaluate infants’ motor development process. The results from the experiments suggest that the proposed system may enable diagnosis support in clinical practice.

## Introduction

Infants acquire motor skills through a development process showing general movements, crawling, standing caught, bipedal walking within the age of about 1 year. Some attempts to quantitatively evaluate the general movements have been reported since the relationship between early movement abnormalities and later developmental disorders was systematically clarified^1^. For example, Taga^2^ performed movement analysis using markers attached to the limbs of the infants and found that the repertoire of the general movements gradually decreases in the early development stage whereas the movements subsequently increases, demonstrating a U-shape. More recently, Nakashima et al.^3^ proposed a markerless movement extraction method using video images, and an evaluation and automatic classification system for general movements. Motor skill pertaining to walking has also been extensively quantified and systematically analyzed^4–7^. For an example, Adolph et al. performed a large-scale walking ability assessment of 225 children in kindergarten.

Meanwhile, although crawling is an important motor skill involving the development of the upper and lower limbs^8^, the relationship between early movement abnormalities and later developmental disorders is not well understood. Some pioneering research on evaluation of crawling started from beginning in the 1990s^9–13^. For example, Segawa et al. focused on the posture of crawling and classified it into six types including belly crawling and hands-and-knees creeping, in which the body is supported by the palms and knees^9^. Adolph et al. carried out a longitudinal study to quantitatively evaluate belly crawling and hands-and-knees creeping by analyzing video images at each frame^11^. Recently, under the hypothesis that the U-shaped development observed in general movements also appears in crawling, Terao et al. analyzed the relationship between the elapsed days since the start of crawling (hereafter referred to as crawling experience) and the number of the repertoire regarding belly crawling and hands-and-knees creeping^14^. However, these studies employed visual observation methods; thus, objective and quantitative evaluation was difficult. Some studies have introduced objective approaches to evaluate infants’ crawling using tracking system with markers^15^, motion capture sensors^16^, surface electromyograms^17^, or goniometers^18^. Such approaches, however, required markers or sensors to be attached to the infant’s bodies; thus, their natural movements may be hindered.

The purpose of this paper is to develop a quantitative and objective crawling evaluation system based on infant movement features that are extracted by a markerless motion extraction method. In the proposed system, infants’ movement features were extracted from video images via image processing, and evaluation indices were calculated based on clinical knowledge. This enabled objective and quantitative evaluation of the crawling movements without the need for attaching markers to the infants’ bodies. The validity of the proposed system to clarify the development process of crawling was verified via cross-sectional and longitudinal analysis of hands-and-knees creeping and belly crawling.

## Methods

### Proposed system

**Figure 1:**
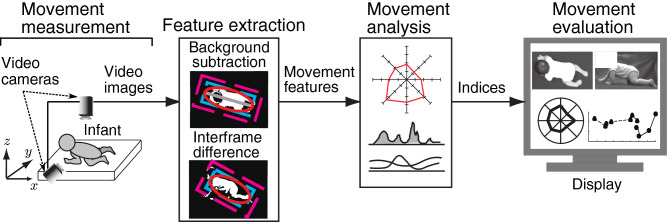
Overview of the proposed system. The system consists of movement measurement, feature extraction, movement analysis, and movement evaluation. The features of infant crawling are extracted from the recorded video images based on image processing, and then evaluation indices based on clinical knowledge are calculated from the movement features.

The configuration of the proposed crawling analysis system is shown in Fig. 1. The proposed system consists of three processes: movement measurement, feature extraction, and movement analysis. The details of the system are described below.

#### Movement measurement

**Figure 2:**
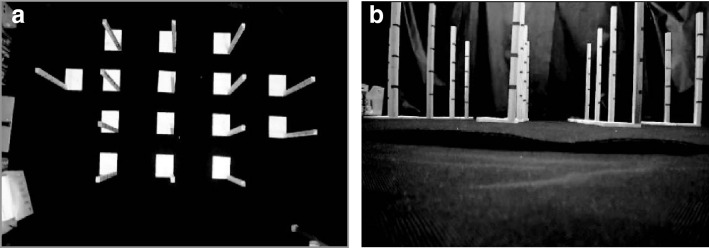
Camera calibration. Reference objects of known three-dimensional shapes are located on the floor. (**a**) Image of ceiling camera. (**b**) Image of lateral camera.

The proposed system concurrently measures movements using two video cameras (HDR-PJ680, Sony Corporation, Tokyo, Japan), one facing down toward the floor (ceiling camera) and the other facing the wall (lateral camera), to record top and lateral views of the infant as shown Fig. 1. Here, the resolution of the cameras is $W \times H$ pixels, and sampling frequency is $f_s$ Hz. For later three-dimensional analysis, the camera is calibrated using 15 wooden sticks placed as shown in Fig. 2 to calculate the actual distances in the recorded images, and black cloths are laid on the floor and wall so that the infant can be extracted from the background color.

#### Feature extraction

**Figure 3:**
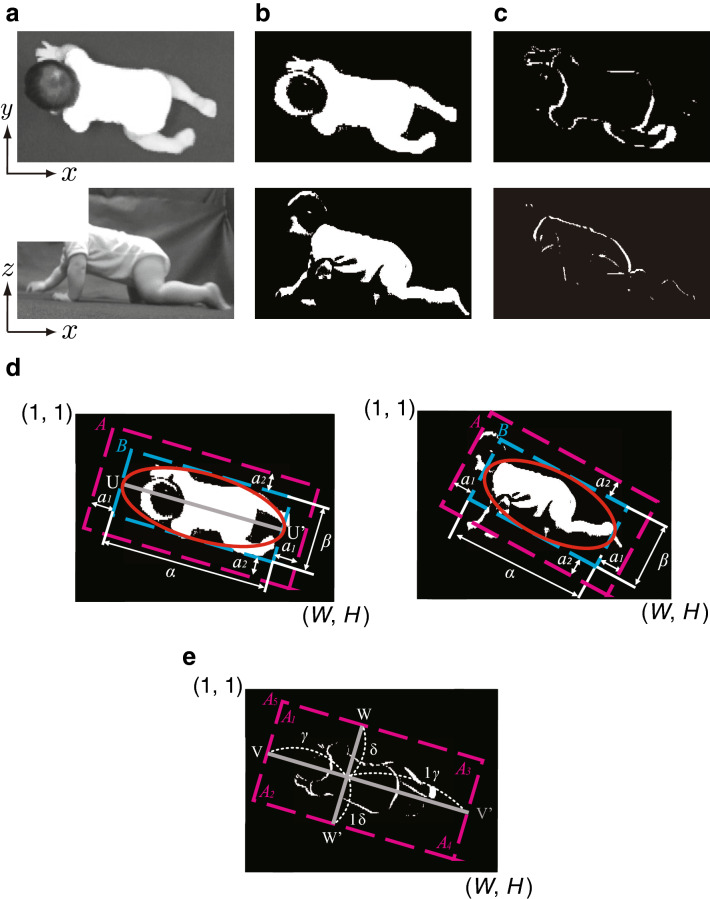
Input images and feature-extracted images. (**a**) Video images measured using ceiling and lateral cameras are converted into grayscale images. (**b**) A background subtraction image is calculated from the difference between a measured video image and the background image with a threshold *T*. (**c**) An inter-frame difference image is calculated by binarizing the subtraction of two neighboring frames with a threshold *T*. (**d**) The outline of the infant body is approximated from the background subtraction image with an ellipse by least squares method. The rectangle (area B) circumscribing the ellipse is also calculated. The lengths of the major and minor axes are denoted by $\alpha$ and $\beta$, respectively, and $a_1$ and $a_2$ are margins to determine the size of the analysis area *A*, so that the infant movements are included in the area. (**e**) Line segments and divided areas. The interframe difference image is divided by two orthogonal line segments into four subareas: $A_1$, upper-right body; $A_2$, upper-left body; $A_3$, lower-right body; and $A_4$, lower-left body. $A_5$ indicates the whole body. The ratio of division is determined by $\gamma$ and $\delta$.

This part extracts movement features from the video images. Fig. 3 shows an example of the captured video image and its feature extraction image. First, the proposed system converts the measured video image (Fig. 3a) into a grey scale component image and into a binary background subtraction image using the threshold *T* (Fig. 3b).

At the same time, an inter-frame subtraction image (Fig. 3c) is generated by differentiating the images between adjacent frames such that a pixel value of 0 (black) indicates no movement detected, and a pixel value of 1 (white) indicates detection of movement binarized by the threshold *T*. Here, to determine the movement area from the video image, the outline of the infant’s body is first approximated from the background subtraction image. An ellipse is fitted for a set of two-dimensional white pixels composing the infant’s body using the least-squares method^19^ implemented in the Intel OpenCV library^20^, and a rectangle circumscribing the ellipse (area *B*) is calculated (Fig. 3d). The numbers of pixels in the long axis and the short axis of the area *B* are respectively represented as $\alpha$ pixels and $\beta$ pixels. Then, margins of $a_1$ pixels and $a_2$ pixels are added to the upper, lower, left, and right sides of area *B* so that the analyzed area *A* and the movement of the infant within the analyzed area can be determined. The margins are determined by $a_1=t_{a_1}\alpha$, $a_2=t_{a_2}\beta$ (where $t_{a_1}$ and $t_{a_2}$ are constants.) in each direction of area *B*. In addition, a line segment VV′ that divides the movement area *A* by ratio of $\gamma :(1-\gamma )$ and a line segment WW′ that divides the movement area B by ratio of $\delta :(1-\delta )$ are determined from the ratio of the size between the upper body and lower body (Fig. 3e). The segmented areas are defined as $A_k$ $(k=1,2,\ldots ,5)$, and, as shown in Fig. 3e, the area corresponding to the right upper limb of the infant is defined as $A_1$; the left upper limb, right lower limb, and left lower limb are respectively defined as $A_2$, $A_3$, and $A_4$, and the whole body is defined as $A_5$.

The differential motion amount (DMA) ${}^{(k)}M_l$ in the area $A_k$ $(k=1,2,\ldots ,5)$ is determined from the inter-frame subtraction image of the ceiling camera by the following equation:

1 $$\begin{aligned} ^{(k)}M_l = \sum _{(x, \ y)\in {A_k}}{O'_l{(x, \ y)}} \end{aligned}$$

where $l \ (l=1, \ 2,\ldots , \ L)$ is the frame number of the video image and *L* is the total number of frames. In addition, $O'_l{(x, \ y)}$ represents the pixel value of the inter-frame subtraction image with respect to (*x*, *y*) $(x=1,\ldots , W; y=1,\ldots ,H$ where the origin (1, 1) is defined as the upper left point in the video image measured by the ceiling camera. $O'_l{(x, \ y)}=1$ represents white pixels, and $O'_l{(x ,\ y)}=0$ represents black pixels. To reduce noise, a low-pass second-order Butterworth filter with cutoff frequency $f'_{\text {low}}$ Hz is applied to $^{(k)}M_l \ (k=1, 2, 3, 4)$, and a second-order bandpass filter with passband $f_{\text {low}}$–$f_{\text {high}}$ Hz is applied to $^{(5)}M_l$.

The image Center of Gravity (CoG) vector, ${{\mathbf {G}}_{l}}$ = $[G^{\text {x}}_l, \ G^{\text {y}}_l, \ G^{\text {z}}_l]$, with spatial coordinates in the real world, is also calculated. First, the CoG coordinates, $\hat{G^{\text {x}}_l}, \hat{G^{\text {y}}_l}, \hat{G^{\text {z}}_l}$, in the camera images are calculated from the background subtraction images (Fig. 3b) using the following equations:

2 $$\begin{aligned} \hat{G^{\text {x}}_l}= \frac{1}{^{(A)}O^{\text {ceiling}}_{l}}\sum _{(x, \ y)\in {A}}{xO^{\text {ceiling}}_l{(x, \ y)}} \end{aligned}$$

3 $$\begin{aligned} \hat{G^{\text {y}}_l} = \frac{1}{^{(A)}O^{\text {ceiling}}_{l}}\sum _{(x, \ y)\in {A}}{yO^{\text {ceiling}}_l{(x, \ y)}} \end{aligned}$$

4 $$\begin{aligned} \hat{G^{\text {z}}_l} = \frac{1}{^{(A)}O^{\text {lateral}}_{l}}\sum _{(x, \ z)\in {A}}{zO^{\text {lateral}}_l{(x, \ z)}} \end{aligned}$$

where $O^{\text {ceiling}}_l (x, y)$ and $O^{\text {lateral}}_l (x, z)$ represent the pixel value of the background subtraction image in the ceiling and lateral camera images, respectively. ${^{(A)}O^{\text {ceiling}}_{l}}$ and ${^{(A)}O^{\text {lateral}}_{l}}$ are the sum of $O_l^{\text {ceiling}} (x,y)$ and $O_l^{\text {lateral}} (x,z)$ in the analyzed area *A*. Using the CoG coordinates vector [$\hat{G^{\text {x}}_l}, \hat{G^{\text {y}}_l}, \hat{G^{\text {z}}_l}$], with camera image coordinates, and solving the corresponding point problem based on the preliminary calibration, the image CoG vector, ${\mathbf {G}}_l=\left[ {G}_l^x, {G}_l^y, {G}_l^z\right]$ with spatial coordinates in the real world is calculated.

It should be noted that since the measurement condition differs for each video image, the margins $t_{a1}$, $t_{a2}$ of the analyzed area and the ratios $\gamma$, $\delta$ of the area division should be set in a way that the movement of each infant is within the analyzed area. The threshold *T* is also established in such a way to extract a background subtraction image properly.

#### Movement analysis

The movement analysis component of the proposed system calculates eight indices from the movement features for the quantitative evaluation of crawling based on clinical knowledge from the extracted DMA $^{(k)}M_{l}$ and image CoG vector ${{\varvec{G}}_{l}}$. The indices are (i) rhythm of movement, (ii) laterality of movement, (iii) cooperativeness of upper limbs, (iv) number of retrogressions in image CoG, (v) standard deviation of image CoG in the medial and lateral directions, (vi) vertical deviation of image CoG, (vii) average speed of image CoG, and (viii) average acceleration of image CoG.

(i) Rhythm of movement: Rhythm of movement is defined by the average frequency $F_\text {M}$ of the power spectral density *P*(*f*) calculated from DMA $^{(5)}M_{l}$ using the autoregressive model:
  5 $$\begin{aligned} F_{\text {M}}=\frac{\displaystyle \sum ^{f_{\text {max}}}_{f=0}fP(f)}{\displaystyle \sum ^{f_{\text {max}}}_{f=0}P(f)} \end{aligned}$$
  where $f = Kf_{\text {s}}/N$ ($K = 0, \ 1, \ \ldots , \ N/2$; *N* is the number of sampling points). If $F_{\text {M}}$ is large, infant crawling includes quick and rhythmic movements.
(ii) Laterality of movement: Laterality of movement is defined by the following equation:
  6 $$\begin{aligned} \theta = \left| \frac{\pi }{4}- \cos ^{-1}\left( \frac{\max _{l} \ ^{(1)}M_l}{\sqrt{ (\max _{l} \ ^{(1)}M_l)^2 + (\max _{l} \ ^{(2)}M_l)^2 } }\right) \right| \end{aligned}$$
  This is the argument from $\pi /4$ of the vector obtained from each maximum value of $^{(1)}M_l$ and $^{(2)}M_l$. Thus, the crawling movement exhibits less laterality as $\theta$ becomes closer to zero, meaning the movement can be performed in a well-balanced manner.
(iii) Cooperativeness of upper limbs: Cooperativeness of the upper limbs is defined by the correlation coefficient *R* between the DMA of the right upper limb and left upper limb:
  7 $$\begin{aligned} R = \frac{\displaystyle {\sum ^{L}_{l=1}}(^{(1)}M_l-^{(1)}{\overline{M}})(^{(2)}M_l-^{(2)}{\overline{M}})}{\sqrt{\displaystyle {\sum ^{L}_{l=1}}(^{(1)}M_l-^{(1)}{\overline{M}})^2}\sqrt{\displaystyle {\sum ^{L}_{l=1}}(^{(2)}M_l-^{(2)}{\overline{M}})^2}} \end{aligned}$$
  where $^{(k)}{\overline{M}}$ is the average value of DMA $^{(k)}M_{l}$ at window width *L*. A positive correlation is obtained when movements of the upper limbs are synchronized on the left and right side. If the upper limbs can be moved alternately on the left and right side as development progresses, a negative correlation is obtained.
(iv) Number of retrogression in image CoG: Number of retrogressions in image CoG is defined by the number of times $r_{\text {T}}$ when ${\mathbf {u}} \cdot ({\mathbf {G}}_{l+1}-{\mathbf {G}}_{l})<0$, where ${\mathbf {u}}$ is the infant’s travel vector calculated from the orthogonal projection of the ellipse of ${\mathbf {G}}_L-{\mathbf {G}}_1$ to the long axis UU’. If crawling is smooth, the number of retrogressions becomes smaller.
(v) Standard deviation of image CoG in the medial and lateral directions: Standard deviation of image CoG in the medial and lateral directions is defined by the standard deviation *S* of the image CoG variance projected onto the direction orthogonal to the second principal component direction of the trajectory ${\{ {{\mathbf {G}}}_l\}}^L_{l=1}$ of $\left( G^{\text {x}}_l, G^{\text {y}}_l \right)$ in the *L* frames:
  8 $$\begin{aligned} S = \sqrt{\frac{1}{L}{\left( \sum ^{L}_{l=1} {\mathbf {E}}_{l}^{\text {T}} {\mathbf {E}}_{l}\right) }} \end{aligned}$$
  where ${\mathbf {E}}_{l}={\mathbf {G}}_{l}-{\mathbf {v}}_{l}$ and ${\mathbf {v}}_{l}$ is the orthogonal projection vector onto the direction of the first principal component of ${\{ {{\mathbf {G}}}_l\}}^L_{l=1}$. Furthermore, ${\mathbf {v}}_{l}$ is calculated using the average vector ${\mathbf {m}}$ of ${\{ {{\mathbf {G}}}_l\}}^L_{l=1}$ and the first principal component vector ${\mathbf {p}}$ by the following equation:
  9 $$\begin{aligned} {\mathbf {v}}_{l}=(({\mathbf {G}}_{l} - \varvec{\mu }) \cdot {\mathbf {p}}){\mathbf {p}} + {\mathbf {m}} \end{aligned}$$
  If an infant sways his or her body to the left and right while crawling, this index, *S*, becomes larger.
(vi) Vertical deviation of image CoG: Vertical deviation of image CoG is defined as the variance $V_{\text {g}}$ of $G^{\text {z}}_l$:
  10 $$\begin{aligned} \bar{G^{\text {z}}}= & {} \frac{1}{L}\sum ^{L}_{l=1}G^{\text {z}}_l \end{aligned}$$
  11 $$\begin{aligned} V_{\text {g}}= & {} \frac{1}{L}\sum ^{L}_{l=1}(G^{\text {z}}_l-\bar{G^{\text {z}}})^2 \end{aligned}$$
  If the upward and downward movements of the body while crawling are large, this index, $V_{\text {g}}$, becomes larger.
(vii) Average speed of image CoG: Average speed of image CoG is defined by the absolute average value $G^{\text {v}}_{\text {ave.}}$ of image CoG speed:
  12 $$\begin{aligned} G^{\text {v}}_{{l}} = f_{\text {s}}\sqrt{(G^{\text {x}}_{l+1}-G^{\text {x}}_{l})^2+(G^{\text {y}}_{l+1}-G^{\text {y}}_{l})^2+(G^{\text {z}}_{l+1}-G^{\text {z}}_{l})^2} \end{aligned}$$
  13 $$\begin{aligned} G^{\text {v}}_{\text {ave.}} = \frac{1}{L-1}\sum ^{L-1}_{l=1}G^{\text {v}}_{{l}} \end{aligned}$$
  This index represents the quickness of movement.
(viii) Average acceleration of image CoG: Average acceleration of image CoG is defined by the absolute average $G^{\text {a}}_{\text {ave.}}$ value of the image CoG acceleration:
  14 $$\begin{aligned} G^{\text {a}}_{{l}} = (G^{\text {v}}_{{l+1}}-G^{\text {v}}_{{l}}) f_{\text {s}} \end{aligned}$$
  15 $$\begin{aligned} G^{\text {a}}_{\text {ave.}} = \frac{1}{L-2}\sum ^{L-2}_{l=1}|G^{\text {a}}_{{l}}| \end{aligned}$$
  This index represents the forcefulness of movement, i.e., the driving force.

In addition, the crawling score *Z* was defined as a relative index for evaluating comprehensive infant development. Initially, indices (v)–(viii) are normalized based on each infant’s height to remove the effect of individual differences in body size. The reciprocals of the values defined in (i), (vi)–(viii) are then calculated as evaluation indices so that the values decrease as development progresses. This is because these indices represent the activeness, forcefulness, and quickness of movement, which are known to increase with developmental progress^12,15^. After that, each evaluation index is standardized with reference to a group of standard developing infants (defined as the control group). This standardization is based on the average value $\mu _j$ $(j = 1, 2,\ldots , 8)$ and standard deviation $\sigma _j$ in the control group as per the following equation:

16 $$\begin{aligned} z_j=\frac{(I_j-\mu _j)}{\sigma _j} \end{aligned}$$

where $I_j$ is the index value before standardization, and the crawling score *Z* is defined as the sum of $z_j$ over the eight evaluation indices. The crawling score represents the degree of difference from the control group, meaning that the higher the score, the higher the probability that motor development is delayed.

#### Experiments

To confirm the effectiveness of the proposed system for the evaluation of infant motor development, crawling analysis was performed on 17 infants. For 16 infants (Participants A–P), measurement was performed once at 10 months after birth. For one infant (Participant Q), measurements were performed 11 times from 7 months to 10 months after birth. Participants A–P were divided into a belly crawling group (4 participants) and a hands-and-knees creeping group (12 participants) based on their Kyoto Scale of Psychological Development^21^ (K-test) results. The K-test is one of the most widely used developmental tests in Japan^22^. The number of items passed among three items (belly crawling, hands-and-knees creeping, and walking) in the results of K-test was calculated for each participant and defined as the K-test score. All measurements were conducted with informed consent from each infant’s parents. All experiments were conducted in accordance with the Declaration of Helsinki and were approved by the Hiroshima University Ethics Committee (Registration number: E-1150-1).

As preprocessing for analysis, the frames in which the whole body of the infant appears were extracted from for each video image. The analysis target section was defined as one straight crawling period (from the landing of one hand on the ground until the same hand lands next) in which $G^{\text {x}}_l$ and $G^{\text {y}}_l$ have a significant correlation. A maximum of three analysis target sections were extracted from each participant, and the number of analysis target sections differed across participants. Ultimately, 34 sections ($L=34.8\pm 12.0$ frames) were extracted in total. The parameters were set as ${f_{\text {s}}} = 30$ Hz, $f'_{\text {low}} = 3$ Hz, $f_{\text {low}} = 0.6$ Hz, $f_{\text {high}} = 3$ Hz, $W=720$ pixel, $H=480$ pixel. The examination of the validity of the index calculation in the proposed system is presented in Supplementary Material. In this examination, the number of retrogressions in image CoG [index (iv)], which is an index that can be judged visually, was compared with the evaluation by an expert (physical therapist).

Infants’ crawling characteristics generally change from belly crawling to hands-and-knees creeping as development progresses^12^. Therefore, we compared the belly crawling group and the hands-and-knees creeping group to confirm whether the proposed system can capture the characteristics of movement while infants develop. In this comparison, the crawling score *Z* was calculated via standardization using the hands-and-knees creeping group (Participants E–P) as a control group.

The data obtained from Participant Q was used to test the ability of the system to evaluate the development process of crawling. Based on each index (i)–(viii) and crawling score *Z* standardized by Participants E–P, the crawling of Participant Q was chronologically analyzed using data recorded on 11 different days among the 67th to the 140th day after crawling movements started. In this experiment, the analysis target was $L=45.4 \pm 18.1$ frames. Each video image was classified by physical therapists as the belly crawling period (crawling experience: 67th to 112th day and 118th day) and hands-and-knees creeping period (117th day and 120th to 140th day).

## Results

**Figure 4:**
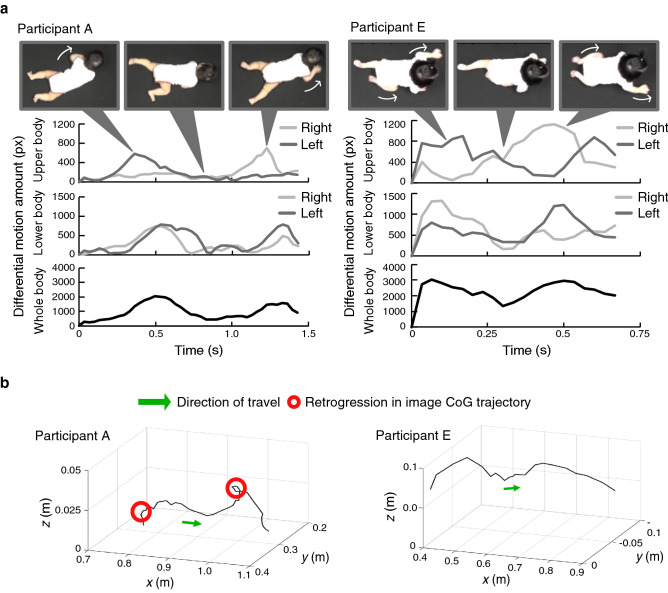
Example of the features of Participant A (belly crawling) and Participant E (hands-and-knee creeping). (a) Time series of differential motion amount for the upper body (right: $^{(1)}M_l$, left: $^{(2)}M_l$), lower body (right: $^{(3)}M_l$, left: $^{(4)}M_l$), and whole body ($^{(5)}M_l$) and corresponding photographs. (b) Trajectories of the image center of gravity. The green arrow and red circle represent the direction of travel and the detected retrogression in the image center of gravity (CoG) trajectory, respectively.

**Figure 5:**
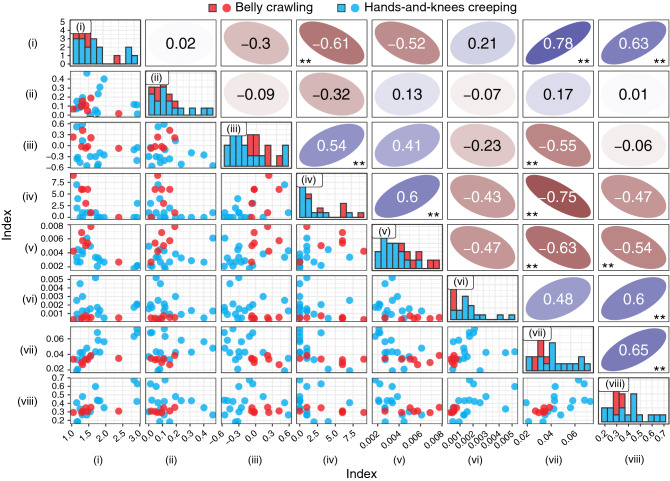
Pair plot of all indices. Diagonal elements represent the histograms of the indices. Lower and upper triangular elements represent the scatterplots for the combinations of each index and the corresponding spearman’s rank correlation coefficients, respectively. Each index represents its raw value (i.e., before reciprocal, normalization, and standardization). The results of the significance tests of the correlation coefficients are also shown (**$p < 0.01$).

The DMA for the upper body (right: $^{(1)}M_l$, left: $^{(2)}M_l$), lower body (right: $^{(3)}M_l$, left: $^{(4)}M_l$), and whole body ($^{(5)}M_l$) are shown in Fig. 4a. The figure includes the results obtained by analyzing Participant A, who had particularly substantial lateral swing and sometimes moved the CoG backwards in the belly crawling group, and Participant E, who was particularly stable in the hands-and-knees creeping group. In addition, Fig. 4b shows the three-dimensional image CoG trajectory of Participants A and E. Figure 5 shows the correlations for each combination of indices as a pair plot. In this figure, each index represents its raw value (i.e., before reciprocal, normalization, and standardization). Diagonal elements represent the histograms of the indices. Lower and upper triangular elements represent the scatterplots for the combinations of each index and the corresponding spearman’s rank correlation coefficients, respectively.

**Figure 6:**
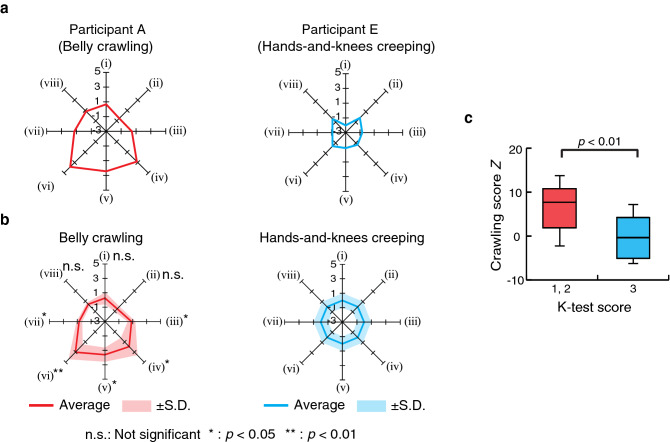
Results of movement analysis. (**a**) Examples of radar charts of the standardized indices for Participant A (belly crawling) and Participant E (hands-and-knee creeping). (**b**) The average radar charts of the standardized indices for belly crawling group and hands-and-knees creeping groups for Participant A–P. Indices (i), (vi)–(viii) have their reciprocal numbers. (**c**) Comparison of crawling score *Z* between participants with different K-test scores. The K-test score is determined by the number of evaluation items that the infant was confirmed to have passed. The three items (belly crawling, hands-and-knee creeping, and walking) were selected from the Kyoto Scale of Psychological Development^21^. The crawling score *Z* is the sum of the eight indices standardized by the hands-and-knees creeping group.

**Figure 7:**
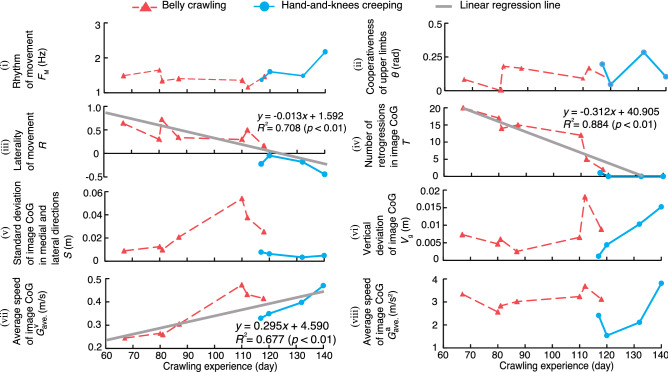
Chronological changes of the evaluation indices for Participant Q.

**Figure 8:**
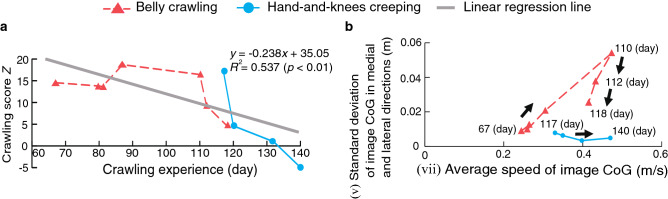
Chronological changes. (**a**) Chronological changes of crawling score Z for Participant Q. (**b**) Chronological change of the relationship between indices (v) and (vii) for Participant Q.

Figure 6a shows radar charts of standardized evaluation indices for Participants A and E. Figure 6b show the average of the standardized indices for the belly crawling group and hands-and-knees creeping group (Participants A–P). An unpaired *t*-test for the average of indices (iii)–(vii) found significant differences ($p<0.05$) between the two groups. Figure 6c shows boxplots of the crawling scores comparing participants with a K-test score of 1 or 2 and those with a K-test score of 3. Figure 7 shows chronological changes of the evaluation indices $I_j$ for Participant Q. A single regression analysis was performed by setting each index value as the objective variable and the crawling experience as the explanatory variable. As a result, a significant regression relationship was observed among cooperativeness of the upper limbs (index (iii)), number of retrogressions in image CoG (index (iv)), and average speed of image CoG (index (vii)). For these indices, the regression equation, the coefficient of determination $R^2$, and the *p* value obtained by *F* test are shown in the figure. Figure 8a shows the chronological change in crawling score according to crawling experience. In addition, chronological changes in the relationship between standard deviation of image CoG in the medial and lateral directions (index (v)) and average speed of image CoG (index (vii)) are also shown in Fig. 8b. In the figure, the vertical axis represents the standard deviation of image CoG in the medial and lateral directions, and the horizontal axis represents the average speed of image CoG.

## Discussion

The movement in one crawling period (from landing of one hand on the ground until the same hand lands again) was extracted by the time series of the DMA $^{(5)}M_l$ showing bimodality in both participants (Fig. 4a). The difference in the duration of one crawling period between Participants A and E indicates the difference in the movement speeds between belly crawling and hands-and-knees creeping. In addition, it can be seen that Participant A (belly crawling) proceeded while moving the left and right lower limbs almost simultaneously, whereas Participant E (hands-and-knee creeping) proceeded while moving the left and right upper and lower limbs alternately. Such differences between participants in the timing of upper and lower limb movements are known as typical differences between belly crawling and hands-and-knee creeping^12^. The circles on the CoG trajectory of Participant A shown in Fig. 4b suggest that the CoG of Participant A moved backwards twice. By contrast, Participant E did not show such a feature, and moved straight forward. These results indicate that the characteristics of crawling by each participant can be extracted from the defined features.

From the pair plot of indices, significant correlations were found between some of the indices (Fig. 5). The index (vii), average speed of image CoG, tends to show relatively high correlation coefficients with other indices, especially with (i) rhythm of movement and (iv) number of retrogression in image CoG, with a positive correlation of 0.78 and a negative correlation of $-\,0.75$, respectively. In addition, the values of such indices for the hands-and-knees creeping group tended to be larger than those for the belly crawling group. These results indicate that infants who have advanced development and were able to crawl at a high speed moved their bodies rhythmically and smoothly. It should be noted that the experiment was performed without attaching sensors or markers to the participants; therefore, it is difficult to directly evaluate the validity of the index calculation in the system through comparison with such physical measurements. Nevertheless, because the features extracted by the proposed system corresponded to the infants’ movements on the video images, as seen from Fig. 4, the indices calculated based on these features are also considered to have a certain degree of reliability. The validity of the index calculation is also partly supported by the finding of the significant association between the expert’s and system’s assessments regarding the retrogression in image CoG (see Supplementary Material).

The standardized indices of Participant A in Fig. 6a shows that indices (ii) and (iii) are large values, which capture the characteristic of belly crawling, where Participant A swayed the body side to side and sometimes moved the CoG backward. By contrast, these indices are small for Participant E (see Fig. 6a, indicating that the crawling was stable. Physical therapists observed that Participant E had propulsive force and moved quickly, and the change of CoG to the medial and lateral directions was small. These observations are consistent with the trend indicated by the indices. The same trends can also be confirmed in the average values of all participants, as shown in Fig. 6b, where the radar chart of the belly crawling group was larger than that of the hands-and-knees creeping group, and statistically significant differences ($p<0.05$) were confirmed in indices (iii)–(vii). These results demonstrate the ability of the proposed index to extract the difference between belly crawling and hands-and-knees creeping. In addition, Fig. 6c shows that the proposed crawling score *Z* of the participants with a K-test score of 3 was significantly smaller than that of the participants with a K-test score of 1 or 2 ($p<0.01$). Therefore, the proposed indices can capture the development stage of crawling from belly crawling to hands-and-knees creeping, and may be applied to evaluating the motor development of infants by associating the indices to the next walking stage.

Longitudinal analysis of Participant Q revealed that cooperativeness of the upper limbs (index (iii)) gradually decreased to 0.44 on the last measurement day (Fig. 7). The moderate negative correlation between the movement of the right upper limb and the left upper limb indicates that Participant Q gradually became able to move the limbs alternately. The decrease in the number of backward movements detected by image CoG (index (iv)) and increase in the average speed of image CoG (index (v)) with the crawling experience indicate that the infant learned efficient movements towards the traveling direction through the process of motor development and acquired rapid motor skill. Regarding the other indices, significant associations with crawling experience changes with crawling experience were not observed, and no significant linear correlation with crawling experience was detected. However, as shown in Fig. 8a, the crawling score decreased with crawling experience. These results suggest that the proposed indices and the crawling score can evaluate changes in the development of crawling over time.

We then focused on the relationship between standard deviation of image CoG in the medial and lateral directions and the average speed of image CoG (Fig. 8b). The standard deviation of image CoG in the medial and lateral directions increased as movement speed increased up to 110 days, but then sharply decreased during the transition period from belly crawling to hands-and-knees creeping. Finally, after the transition to hands-and-knees creeping, the average speed increased, while the standard deviation of image CoG became small. This change in time course suggests that the infant acquired stable crawling skill through the development process from belly crawling to hands-and-knees creeping. This analysis result suggests that the proposed system enables the evaluation of change in the development of crawling by analyzing the relationship between the evaluation indices.

In conclusion, we propose a crawling analysis system that enables the quantitative and objective evaluation of crawling without markers attached to the infants. This system measures infant movements using two video cameras and extracts movement features represented by eight indices through image processing. Cross-sectional analysis of 16 participants demonstrated that the proposed system could extract characteristics of crawling for each participant and quantitatively evaluate both belly crawling and hands-and-knees creeping based on the proposed evaluation indices. We also demonstrated the possibility of associating the indices with infant motor development by comparing crawling scores with K-test scores. Longitudinal analysis of one participant demonstrated that the proposed system can evaluate chronological changes in crawling, indicating the possibility of quantitative evaluation of the crawling development process.

In the longitudinal analysis, only one stable crawling bout (defined as one straight crawling period) could be taken on each day due to the limitations of the recording environment. However, bout-level features, such as variability in the indices across multiple crawling bouts on each day may also change as development progresses. Therefore, further analysis should be carried out in the future after improving the recording environment and experimental tasks. We also plan to evaluate other forms of crawling, such as hitching and scooting^10,12,23^. Moreover, in this study, the experimental evaluation was only performed for participants with typical development. We will therefore conduct analysis for infants with developmental disorders and attempt to support early diagnosis based on quantitative evaluation of crawling movements.

## Electronic supplementary material

Supplementary material 1 (PDF 215 kb)

## Data Availability

The videos of Participant Q and codes used in the analysis are available in the figshare repository: 10.6084/m9.figshare.12519893. Other datasets generated and/or analysed in the current study are not publicly available but are available from the corresponding author upon reasonable request.
