# Supplementary material for: Video-based evaluation of infant crawling toward quantitative assessment of motor development
Source: Sci Rep. 2020 Jul 9;10:11266. doi: 10.1038/s41598-020-67855-0 (PMC7347929; doi:10.1038/s41598-020-67855-0)
Supplement: Supplementary file 1 — Supplementary material 1 (PDF 215 kb) [file 41598_2020_67855_MOESM1_ESM.pdf]

Supplementary material

Video-based Evaluation of Infant Crawling toward Quantitative Assessment of Motor Development

Katsuaki Kawashima[1], Yasuko Funabiki[2], Shino Ogawa[2], Hideaki Hayashi[3], Zu Soh[1], Akira Furui[1], Ayumi Sato[2], Taiko Shiwa[2], Hiroki Mori[4], Koji Shimatani[5], Haruta Mogami[2], Yukuo Konishi[6], and Toshio Tsuji[1,*]

[1] Graduate School of Engineering, Hiroshima University, 1-4-1 Kagamiyama, Higashi-Hiroshima, Hiroshima 739-8527, Japan

[2] Graduate School of Human and Environmental Studies, Kyoto University, Yoshida-Nihonmatsu-Cho, Sakyo-Ku, Kyoto, Kyoto 606-8507, Japan

[3] Faculty of Information Science and Electrical Engineering, Kyushu University, 744 Motooka, Nishi-Ku, Fukuoka, Fukuoka 819-0395, Japan

[4] Future Robotics Organization, Waseda University, 1-104 Totsukamachi, Shinjuku-Ku, Tokyo 169-8050, Japan 5Department of Physical Therapy, Prefectural University of Hiroshima, 1-1 Gakuen, Mihara, Hiroshima 723-0053, Japan

[6] Doshisha University Center for Baby Science, 4-1-1 Kizugawadai, Kizugawashi, Kyoto 619-0225, Japan

Validity of the evaluation index

To examine the validity of the index calculated by the proposed system, the number of retrogressions in image CoG [index (iv)], which is an index that can be judged visually, was compared with the evaluation by an expert (physical therapist). The videos of Participants A−P were used for this investigation. For each video, a section with one crawling cycle, which is the same section as the analysis target of the system, was presented to the experts. Because it was difficult visually to count the exact number of retrogressions from a short video of one crawling cycle, the expert was asked to determine whether retrogressions were confirmed or not. We then compared the evaluation results of the expert with those of the system for each video. Here, the judgement of the presence of the retrogressions in the system was defined as the case where the index takes > 0.

Table S1 shows the result of comparing the system's and the expert's assessments of the retrogression in image CoG. Accuracy, sensitivity, and specificity from the confusion matrix were 0.783, 0.765, and 0.833, respectively. Fisher’s exact test revealed a significant association between the expert assessment and system assessment (p = 0.0183). Yule's coefficient of association was 0.884, which means the association is strong. These results partly support the validity of the index calculation in the proposed system.

Supplementary Table S1. Confusion matrix for the assessment of with/without retrogression.

		Expert assessment	
		w/ retrogression	w/o retrogression
System assessment	w/ retrogression	13	1
	w/o retrogression	4	5
